# Antiinfektive Therapie der periprothetischen Infektion: Kurzfassung der AE-Handlungsempfehlung (2026)

**DOI:** 10.1007/s00132-026-04851-8

**Published:** 2026-09-11

**Authors:** Susanne Feihl, S. Meller, Y. Gramlich, R. von Eisenhart-Rothe, R. Hube, P. Radünzel, R. Otchwemah, Gunnar Hischebeth

**Affiliations:** 1 Antimicrobial Stewardship, Technische Universität München, TUM School of Medicine and Health, TUM Klinikum, Ismaninger Straße 22, 81675 München, Deutschland; 2 Centrum für Muskuloskeletale Chirurgie, Charité-Universitätsmedizin Berlin, Berlin, Deutschland; 3 Klinik für Orthopädie und Unfallchirurgie, AGAPLESION Markus Krankenhaus, Frankfurter Diakonie Kliniken, Frankfurt am Main, Deutschland; 4 Klinik für Orthopädie und Sportorthopädie, Technische Universität München, TUM School of Medicine and Health, TUM Universitätsklinikum, München, Deutschland; 5 Orthopädische Chirurgie München (OCM), München, Deutschland; 6 Klinik für Infektiologie und Intensivmedizin, Charité-Universitätsmedizin Berlin, Berlin, Deutschland; 7 Abteilung für Hygiene und Umweltmedizin, Universitätsmedizin Essen, Universität Duisburg-Essen, Essen, Deutschland; 8 Institut für Hygiene, Kliniken Stadt Köln gGmbH, Klinikum Köln-Merheim, Lehrstuhl für Hygiene und Umweltmedizin, Universität Witten/Herdecke, Köln, Deutschland; 9 Klinik für Orthopädie, Unfallchirurgie und Sporttraumatologie, Kliniken Stadt Köln gGmbH, Klinikum Köln-Merheim, Lehrstuhl für Orthopädie und Unfallchirurgie, Universität Witten/Herdecke, Köln, Deutschland; 10 Institut für Medizinische Mikrobiologie, Immunologie und Parasitologie (IMMIP), Universitätsklinikum Bonn, Venusberg-Campus 1, 53127 Bonn, Deutschland

**Keywords:** Antiinfektiva, Biofilm, Konsens, Medikamentennebenwirkungen, Gelenkprothesen, Antiinfective agents, Biofilm, Consensus, Drug side effects, Joint prosthesis

## Abstract

**Hintergrund:**

Periprothetische Infektionen (PPI; engl.: PJI) zählen zu den schwerwiegendsten und komplexesten Komplikationen nach endoprothetischem Gelenkersatz und erfordern ein strukturiertes interdisziplinäres Therapiekonzept. Neben einer adäquaten chirurgischen Strategie kommt insbesondere einer möglichst erregergerichteten sowie patientenindividuell angepassten antiinfektiven Therapie eine zentrale Bedeutung zu.

**Methoden:**

Auf Grundlage einer umfassenden Literatur- und Leitlinienrecherche sowie einer anschließenden Konsensbildung innerhalb der interdisziplinären Arbeitsgruppe der „Deutschen Gesellschaft für Endoprothetik e. V.“ wurden bestehende Therapieoptionen bewertet und konsentierte Handlungsempfehlungen zur antiinfektiven Therapie periprothetischer Infektionen erarbeitet.

**Ergebnisse:**

Die Basis der Behandlung periprothetischer Infektionen bildet die chirurgische Sanierung in Kombination mit einer initial kalkulierten und anschließend gezielten Antibiotikatherapie. Staphylokokken stellen die häufigsten Erreger dar; hierbei kommt dem biofilmwirksamen Antibiotikum Rifampicin eine wesentliche Rolle zu. Fluorchinolone werden insbesondere bei Infektionen mit gramnegativen Erregern eingesetzt, finden jedoch auch als Kombinationspartner von Rifampicin bei Infektionen die durch Staphylokokken verursacht sind Anwendung. Für weitere klinisch relevante Erregergruppen wurden differenzierte konsentierte Therapieempfehlungen formuliert. Die empfohlene Gesamtdauer der antibiotischen Therapie beträgt in der Regel etwa 12 Wochen.

**Schlussfolgerung:**

Die antiinfektive Therapie ist eine wichtige Säule für die erfolgreiche Therapie der periprothetischen Infektion. Ein individualisiertes, an das chirurgische Behandlungskonzept angepasstes Vorgehen ist dabei erforderlich.

## Abstract (en)

**Background:**

Periprosthetic joint infections (PJI) are among the most severe and complex complications following joint arthroplasty and require a structured interdisciplinary treatment approach. In addition to an adequate surgical strategy, pathogen-directed and patient-specific anti-infective therapy plays a central role in successful management.

**Methods:**

Based on a comprehensive literature review and current guidelines, followed by a structured consensus process within the interdisciplinary working group of the “German Society for Arthroplasty (Deutsche Gesellschaft für Endoprothetik e. V.)”, available treatment options were evaluated and consensus recommendations for the anti-infective management of PJI were developed.

**Results:**

The cornerstone of PJI treatment is surgical management combined with an initial empiric followed by targeted antibiotic therapy. Staphylococci represent the most frequently identified pathogens, with the biofilm-active antibiotic rifampin playing a key role in their treatment. Fluoroquinolones are primarily used for infections caused by gram-negative pathogens but are also employed in combination with rifampin for staphylococcal infections. For other clinically relevant pathogen groups, differentiated consensus-based treatment recommendations were established. The recommended total duration of antibiotic therapy is generally approximately 12 weeks.

**Conclusion:**

Anti-infective therapy represents a key component in the successful treatment of periprosthetic joint infections. An individualized therapeutic approach, tailored to the respective surgical treatment strategy, is essential.

## Einleitung

Für die erfolgreiche Behandlung der periprothetischen Infektion ist ein strukturiertes Vorgehen notwendig. Neben der Erregerdiagnostik und der chirurgischen Therapie spielt die antiinfektive Therapie eine wichtige Rolle. Das Behandlungskonzept soll den Erreger gezielt erfassen und auf die Patienten individuell abgestimmt werden.

## Das chirurgische Behandlungskonzept

Die chirurgische Therapie bildet die Grundlage der Behandlung von PPI, sofern der Allgemeinzustand der betroffenen Patienten eine Operation erlaubt. Die chirurgische Therapie richtet sich dabei nach der Dauer der Beschwerden, sowie patientenindividuellen und infektiologischen Gesichtspunkten. Grundsätzlich lassen sich zwei operative Ansätze unterscheiden:

1. Prothesenerhaltende Verfahren:
  Diese beinhalten ein subtiles, teils radikales Debridement mit Austausch der modularen, beweglichen Prothesenkomponenten. Dieses Verfahren wird auch als DAIR-Prozedur bezeichnet. DAIR steht dabei für Débridement, „antibiotics“ (Antibiothikatherapie), „irrigation“ (Spülung) und „implant retention“.
2. Prothesenwechsel:
  Dabei wird das gesamte Implantat und weiteres Fremdmaterial entfernt und eine neue Endoprothese implantiert. Wechseloperationen können einzeitig (Explantation und Reimplantation in einer Sitzung) oder mehrzeitig (Ex- und Reimplantation in separaten Eingriffen) erfolgen.
  Bei mehrzeitigen Wechseln wird häufig ein antibiotikahaltiger Zement-Spacer temporär implantiert. Im prothesenfreien Intervall wird eine biofilmwirksame Therapie mit Rifampicin in der Regel vermieden. Die Dauer des prothesenfreien Intervalls vor Reimplantation variiert: Ein „kurzes Intervall“ endet nach ca. 2 Wochen, während ein „langes Intervall“ 6 Wochen oder mehr umfasst.

Die antibiotische Therapie ist eng mit dem chirurgischen Konzept verknüpft. Nach der Durchführung des chirurgischen Debridements ist eine antibiotische Behandlung indiziert. Bei prothesenerhaltenden Verfahren sowie nach Reimplantation der neuen Endoprothese im Rahmen von Wechseloperationen erfolgt i. d. R. eine gezielte biofilmaktive Therapie. Anmerkung: die perioperative Antibiotikaprophylaxe soll stets verabreicht werden, auch bei geplanter Probenentnahme und ist in der S3-Leitlinie *Perioperative und Periinterventionelle Antibiotikaprophylaxe* (2024) geregelt [1].

## Empirische und erregerspezifische Therapie

Zu Beginn der Therapie bzw. direkt postoperativ liegt häufig noch kein Erregernachweis vor. Die antiinfektive Behandlung wird deshalb zunächst empirisch (kalkuliert) ohne Zeitverzögerung gestartet. Die Auswahl der Substanzen für die empirische Therapie ist abhängig von der klinischen Präsentation, patienteneigenen Risikofaktoren, der lokalen Resistenzsituation und der Vorgeschichte der Patienten. Falls keine Allergie vorliegt wird häufig Ampicillin/Sulbactam mit oder ohne Vancomycin (oder Daptomycin) verabreicht ([25]; Tab. 2).

Nach Erhalt der mikrobiologischen Ergebnisse erfolgt eine Umstellung der kalkulierten Therapie auf eine erregerspezifische Therapie unter Berücksichtigung der Resistenztestung (Tab. 3).

### Staphylokokken

Häufigster Erreger bei PPI sind Staphylokokken. Klassischerweise werden bei Oxacillin-sensiblen Staphylokokkenisolaten Gruppe-1-Cephalosporine (Cefazolin) oder Isoxazolylpenicilline (Flucloxacillin) eingesetzt [21, 33]. Eine durch die Patienten angegebene Penicillinallergie sollte eingehend hinterfragt werden und die Wahl des Antibiotikums unter Berücksichtigung des Reaktionstyps und möglicher Kreuzreaktionen erfolgen [14]. Infektionen durch Oxacillin-resistente Staphylokokken können mit Vancomycin oder Daptomycin adressiert werden. Im Rahmen des DAIR-Konzepts werden die o. g. Substanzen mit Rifampicin kombiniert, sofern keine Resistenz vorliegt. Ziel ist hierbei die Biofilmaktivität dieser Substanz gegen Staphylokokken zu nutzen. Bei Gabe von Rifampicin sollte auf die Wechselwirkungen mit Begleitmedikation geachtet werden, die durch Induktion von CYP3A4-Enzymen in der Leber ausgelöst werden (Tab. 1). Die Therapie mit Rifampicin sollte nach chirurgischer Sanierung möglichst zügig begonnen werden („run for the surface“), jedoch im Kontext einzelner Publikationen ggf. möglichst bei trockenen Wundverhältnissen und nach Entfernung der Drainagen [2, 5]. Staphylokokken mit Rifampicin-Resistenz werden auch als „Problemerreger“ bezeichnet, aufgrund der fehlenden biofilmaktiven Therapiemöglichkeit. Die orale Sequenztherapie sollte mit gut bioverfügbaren Antibiotika durchgeführt werden. Insbesondere sind hier die Gruppe der Chinolone (z. B. Levofloxacin) zu nennen. Der Einsatz sollte unter entsprechender Abwägung der Nebenwirkungen/Wechselwirkungen indiziert werden. Da inzwischen einige Rote-Hand-Briefe bezüglich der Chinolone vorliegen, sollte eine Patientenaufklärung über etwaige Nebenwirkungen/Wechselwirkungen unbedingt erfolgen (Tab. 1). Ist die Therapie mit Chinolonen nicht möglich, kommen Cotrimoxazol, Doxycyclin, Clindamycin und Linezolid in Betracht. Alle Substanzen sollten, wenn möglich, mit Rifampicin kombiniert werden.

**Tab. 1:** Praxistipps zu Nebenwirkungen und zum Monitoring häufig eingesetzter Antiinfektiva

Substanz	Monitoring	Nebenwirkungen/Anmerkungen
Vancomycin	Nierenfunktion, Talspiegel (ca. 2–3 × pro Woche, abhängig von Nierenfkt.)	Niereninsuffizienz, Vancomycin-Infusionssyndrom
Cotrimoxazol	Nierenfunktion, Blutbild, Elektrolyte	Knochenmarkdepression, Hautausschlag, Mukositis
Chinolone (z. B. Levofloxacin, Ciprofloxacin)	Nierenfunktion, QTc-Zeit	Erhöhtes Risiko für Aortenaneurysmata/-dissektion; Tendinitis, psychische Alterationen, periphere Neuropathie, Krampfanfall/Senkung der Krampfschwelle, QTc-Zeit-Verlängerung möglich. Bei peroraler Gabe verminderte Resorption aus Magen-Darmtrakt durch 2- oder 3-wertige Kationen (z. B. Ca, Mg, Fe, Zn in Arzneimitteln oder Lebensmitteln) (siehe Rote-Hand-Briefe)
Doxycyclin	Nierenfunktion, Blutbild, Leberwerte bei längerer Gabe	Sonnenexposition vermeiden. Bei peroraler Gabe verminderte Resorption aus Magen-Darmtrakt durch 2- oder 3-wertige Kationen (z.B. Ca, Mg, Fe, Zn in Arzneimitteln oder Lebensmitteln)
Betalaktamantibiotika (z. B. Ampicillin/Sulbactam, Cefazolin)	Blutbild	Leukopenie, Allergie
Rifampicin	Transaminasen, Interaktionen mit Begleitmedikation prüfen	Verfärbung von Körperflüssigkeiten, Wechselwirkungen mit einer Vielzahl von Medikamenten die über CYP3A4 verstoffwechselt werden. Keine Monotherapie!
Daptomycin	Kreatinkinase	Eosinophile Pneumonie, Rhabdomyolyse
Linezolid	Blutbild, Begleitmedikation prüfen (hinsichtlich serotonergem Wirkstoff)	Myelosuppression, Neurotoxizität, Serotonin-Syndrom; nach Fachinformation Therapiedauer max. 28 Tage
Metronidazol	Blutbild	Knochenmarksdepression, neurologische Nebenwirkungen, Geschmacksstörung (z. B. metallischer Geschmack); nach Fachinformation Therapiedauer max. 10 Tage
Aminoglycoside (z. B. Gentamicin, Tobramycin)	Nierenfunktion, Talspiegel	Niereninsuffizienz, Ototoxizität Keine Monotherapie!
Fluconazol	Leberwerte, QTc-Zeit, Interaktionen mit Begleitmedikation prüfen	QTc-Zeit-Verlängerung möglich

**Tab. 2:** Kalkulierte Antiinfektivatherapie bei periprothetischer Infektion^a^

	Antiinfektivum^b,c^ (Patientenindividuelle Kontraindikationen/Risikofaktoren beachten z. B. Allergien)	Dosierung^c^ (Ggf. Anpassung an Organfunktionen, Alter oder Körpergewicht)
*Ohne Risikofaktoren*		
–	Ampicillin/Sulbactam i.v. oder	3–4 × 3 g
	Cefuroxim i.v.	3 × 1,5 g
*Mit Risikofaktoren*		
lokal hoher Anteil an Oxacillin-Resistenz bei Staphylococcus spp., MRSA-Kolonisation, multiple Voroperationen, schwere systemische Infektion	Ampicillin/Sulbactam i.v. oder	3–4 × 3 g
	Cefuroxim i.v.	3 × 1,5 g
	+	
	Vancomycin i.v.^e^ oder	2 × 15 mg/kg/KG
	Daptomycin i.v.^g^	1 × 8–10 mg/kg/KG
Zusätzlich Risiko für Infektion mit gramnegativen Erregern	Piperacillin/Tazobactam	3–4 × 4,5 g
	+	
	Vancomycin i.v.^e^ oder	2 × 15 mg/kg/KG
	Daptomycin i.v.^g^	1 × 8–10 mg/kg/KG

^a^Gezielte Therapie nach Erreger und Resistenz (siehe Tab. 3)

^b^Allergien gegenüber Antiinfektiva unbedingt berücksichtigen

^c^Angegebene Substanzen und Dosierungen gelten für Erwachsene mit normaler Organfunktion und normalem Körpergewicht; ggf. Anpassung an Kontraindikationen (z. B. Schwangerschaft, Stillzeit), patienteneigene Risikofaktoren, Organfunktion, Körpergewicht und Alter notwendig

^e^Angegebene Vancomycin-Dosierung gilt für Patienten mit normaler Nierenfunktion. Regelmäßige Kontrolle der Nierenwerte und des Talspiegels (Zielwert: 15–20 mg/l) und entsprechende Anpassung der Dosierung notwendig.

^g^Cave Off-Label-Dosierung

*MRSA* Methicillin-resistenter Staphylococcus aureus

**Tab. 3:** Erregerspezifische Antiinfektivatherapie bei periprothetischer Infektion

	Antiinfektivum^b,c^ (Keimspektrum/Resistenz und patientenindividuelle Kontraindikationen/Risikofaktoren beachten)	Dosierung^c^ (Ggf. Anpassung an Organfunktionen, Alter oder Körpergewicht)
**Staphylococcus** **spp.**		
*Initiale Therapie*^*f*^		
Methicillin/Oxacillin-sensibel	Cefazolin oder	3 × 2 g i.v.
	Flucloxacillin	4–6 × 2 g i.v.
Methicillin/Oxacillin-resistent	Vancomycin^e^ oder	2 × 15 mg/kg/KG i.v.
	Daptomycin^g^	1 × 8–10 mg/kg/KG ^g^ i.v.
*Orale Folgetherapie bei einliegendem FM*	Levofloxacin oder	2 × 500 mg p.o.
	Cotrimoxazol oder	3 × 960 mg p.o.
	Doxycyclin	2 × 100 mg p.o.
	+	
	Rifampicin^f^	2 × 450 mg p.o.
Rifampicin-Resistenz	Individuelles Therapiekonzept/ggf. Suppressionstherapie	–
*Orale Folgetherapie im prothesenfreien Intervall/Spacer*	Cotrimoxazol oder	3 × 960 mg p.o.
	Doxycyclin oder	2 × 100 mg p.o.
	Clindamycin	3 × 600 mg p.o.
	Kein Rifampicin	–
**Streptococcus spp.**^d^		
*Initiale Therapie*	Penicillin G oder	4 × 5 Mio. IE i.v.
	Ceftriaxon	1(–2) × 2 g i.v.
*Orale Folgetherapie*	Amoxicillin oder	3(–4) × 1 g p.o.
	Clindamycin	3 × 600 mg p.o.
**Enterococcus spp.: **Penicillin/Ampicillin-sensibel		
*Initiale Therapie*	Ampicillin	4 × 2–3 g i.v.
	± Ceftriaxon oder	2 × 2 g i.v.
	Gentamicin^k^	1 × 3 mg/kg/KG i.v. (max. 1 × 240 mg)
*Orale Folgetherapie*	Amoxicillin	3(–4) × 1 g p.o.
**Enterococcus spp.: **Penicillin/Ampicillin-resistent		
*Initiale Therapie*	Vancomycin^e^ oder	2 × 15 mg/kg/KG i.v.
	Daptomycin^g,l^	1 × 10–12 mg/kg/KG^g^ i.v.
*Orale Folgetherapie*	Linezolid^h^	2 × 600 mg p.o.
Vancomycin-resistent (VRE)	Individuelles Therapiekonzept	–
**Enterobacterales**		
*Initiale Therapie*	Ampicillin/Sulbactam oder	3–4 × 3 g i.v.
	Piperacillin/Tazobactam oder	3–4 × 4,5 g i.v.
	Ceftriaxon	1–2 × 2 g i.v.
*Orale Folgetherapie*	Ciprofloxacin^i^	2 × 500–750 mg p.o.
**Nonfermenter **(z. B. Pseudomonas aeruginosa, Acinetobacter baumannii^j^*)*		
*Initiale Therapie*	Piperacillin/Tazobactam oder	4 × 4,5 g i.v.
	Ceftazidim oder	3 × 2 g i.v.
	Meropenem (A. baumannii^j^*)*	3 × (1–)2 g i.v.
	± Tobramycin^k^	1 × 3(–5) mg/kg/KG i.v.
*Orale Folgetherapie*	Ciprofloxacin^i^	2 × (500–)750 mg p.o.
Ciprofloxacin- oder Multiresistenz	Individuelles Therapiekonzept	***–***
**Anaerobier **(Cutibacterium acnes)		
*Initiale Therapie*	Penicillin G oder	4 × 5 Mio. I.E. i.v.
	Clindamycin oder	3 × 600 mg i.v.
	Vancomycin^e^	2 × 15 mg/kg/KG i.v.
*Orale Folgetherapie bei einliegendem FM*	Amoxicillin	3(–4) × 1 g p.o.
	± Rifampicin^f,m^ oder	2 × 450 mg p.o.
	Levofloxacin	2 × 500 mg p.o.
	+ Rifampicin^f,m^ oder	2 × 450 mg p.o.
	Clindamycin	3 × 600 mg p.o.
*Orale Folgetherapie im prothesenfreien Intervall/Spacer*	Amoxicillin oder	3(–4) × 1 g p.o.
	Clindamycin	3 × 600 mg p.o.
**Grampositive Anaerobier**^**m**^ (nicht Cutibacterium acnes*)*		
*Initiale Therapie*	Ampicillin/Sulbactam oder	3 × 3 g i.v.
	Penicillin G	4 × 5 Mio. I.E. i.v.
*Orale Folgetherapie*	Amoxicillin oder	3(–4) × 1 g p.o.
	Clindamycin	3 × 600 mg p.o.
**Gramnegative Anaerobier**		
*Initiale Therapie*	Ampicillin/Sulbactam oder	3–4 × 3 g i.v.
	Piperacillin/Tazobactam	3–4 × 4,5 g i.v.
*Orale Folgetherapie*	Metronidazol^n^	3 × 400 mg p.o.
**Candida spp.**		
*Initiale Therapie*	Caspofungin oder	Startdosis 1 × 70 mg i.v.; ab 2. Tag 1 × 50 mg^o^ i.v.
	Anidulafungin	Startdosis 1 × 200 mg i.v.; ab 2. Tag 1 × 100 mg i.v.
*Orale Folgetherapie*	Fluconazol (falls sensibel) individuelles Therapiekonzept/ggf. Suppressionstherapie	1 × 400 mg p.o.
**Kultur-negativer Infekt**		
*Initiale Therapie*	Tab. 2	–
*Orale Folgetherapie bei einliegendem FM*	Levofloxacin	2 × 500 mg p.o.
	+ Rifampicin^f^	2 × 450 mg p.o.
*Orale Folgetherapie* *Therapie im prothesenfreien Intervall/Spacer*	Cotrimoxazol	3 × 960 mg p.o.
	+ Amoxicillin (Kombinationstherapie) oder	3 × 1 g p.o.
	Doxycyclin (Monotherapie)	2 × 100 mg p.o.

*FM* Fremdmaterial, *EUCAST* European Commitee on Antimicrobial Susceptibility Testing, *KG* Körpergewicht

^b^Allergien gegenüber Antiinfektiva unbedingt berücksichtigen

^c^Angegebene Substanzen und Dosierungen gelten für Erwachsene mit normaler Organfunktion und normalem Körpergewicht; ggf. Anpassung an Kontraindikationen (z. B. Schwangerschaft, Stillzeit), patienteneigene Risikofaktoren, Organfunktion, Körpergewicht und Alter notwendig (siehe auch Tab. 1)

^d^Limitierte Evidenz für orale Therapie und Betalaktamallergie; mögliche orale Alternativen zu Clindamycin sind Doxycyclin p.o. oder Levofloxacin p.o.; allerdings für Viridans-Streptokokken keine Breakpoints nach EUCAST für Doxycyclin und Levofloxacin

^e^Angegebene Vancomycin-Dosierung gilt für Patienten mit normaler Nierenfunktion. Regelmäßige Kontrolle der Nierenwerte und des Talspiegels (Zielwert: 15–20 mg/l) und entsprechende Anpassung der Dosierung notwendig

^f^Rifampicin erst dazugeben nach Implantation des Fremdmaterials sowie chirurgischer Sanierung sowie bei trockener Wunde. Dosisreduktion auf Rifampicin 2 × 300 mg p.o. bei älteren Patienten (> 75 Jahre). Interaktion mit Begleitmedikation überprüfen sowie Leberwerte (GOT und GPT) monitoren (Tab. 1)

^g^Cave Off-Label-Dosierung

^h^Anwendungsbeschränkung auf 28 Tage (Tab. 1).

^i^Bei Vorliegen einer Ciprofloxacin-Resistenz individuelles Therapiekonzept (siehe auch Text zu gramnegative Erreger)

^j^Bei A. baumanii keine Breakpoints für Piperacillin/Tazobactam und Ceftazidim nach EUCAST, deshalb initiale i.v. Therapie i. d. R. Meropenem

^k^Während Gentamicin- und Tobramycin-Gabe Nephrotoxizität und Ototoxizität beachten. Dosierung gemäß KG, Nierenfunktion und Talspiegel. Regelmäßige Kontrolle der Nierenfunktion und Gentamicin/Tobramycin-Talspiegel

^l^Aufgrund unzureichender Datenlage nach EUCAST keine Breakpoints für Enterococcus spp. verfügbar

^m^In der Literatur keine einheitlichen Angaben bzgl. Zugabe von Rifampicin (siehe auch Text zu Anaerobier)

^n^Nach Fachinformation Therapiedauer auf 10 Tage begrenzt

^o^Bei KG > 80 kg Caspofungin fortführen mit 1 × 70 mg i.v. pro Tag

**Disclaimer:** Für die antiinfektive Therapie der PPI existiert in der Literatur für einige Erreger nur eine limitierte Evidenz. Manuskript und Tabellen wurden mit äußerster Sorgfalt erstellt und erheben keinen Anspruch auf Vollständigkeit. Sie stellen eine praxisorientierte Übersicht dar und ersetzen nicht die individuelle ärztliche Beurteilung, Fachinformation oder aktuelle Leitlinien/Literatur. Im Zweifelsfall Quellen oder Literatur erneut überprüfen. Von den Autoren wird keine Haftung übernommen.

Für den einzeitigen Wechsel gelten bei Staphylokokkeninfektionen die gleichen Empfehlungen wie für das DAIR-Verfahren.

Im Rahmen des zweizeitigen Wechsels gelten ebenfalls die gleichen Grundsätze wie für das DAIR-Konzept bzw. wie für den einzeitigen Wechsel, allerdings im prothesenfreien Intervall ohne Gabe von Rifampicin. Im prothesenfreien Intervall steht die Behandlung der Osteitis/Osteomyelitis im Vordergrund. Die orale Sequenztherapie wird mit den o. g. Substanzen durchgeführt. Die Chinolone sollten allerdings für die Zeit nach der Reimplantation geschont werden.

### Streptokokken

In ca. 10 % der PPI werden Streptokokken detektiert [28, 32]. Falls keine Allergie vorliegt, werden PPI mit Streptokokken in der Regel mit einem Betalaktamantibiotikum therapiert [15]. In Empfehlungen zur antibiotischen Therapie der Streptokokken PPI werden für die intravenöse Therapie z. B. Penicillin G oder Ceftriaxon [21, 25, 33] und u. a. eine orale Sequenztherapie mit Amoxicillin aufgeführt [15, 21, 25, 33]. Einige Studien berichten über einen weniger guten Behandlungserfolg nach einer Infektion mit Streptokokken [4, 10, 18]. Deshalb wird auch eine längere Behandlungsdauer im Sinne einer anschließenden Suppressionstherapie diskutiert [7, 24].

### Anaerobier

Bei chronischen implantatassoziierten Infektionen wird *Cutibacterium acnes* als häufiger Vertreter grampositiver Anaerobier detektiert [3, 23]. Die Empfehlungen zur antibiotischen und insbesondere zur oralen Folgetherapie durch *Cutibacterium acnes* verursachten implantatassoziierten Infektion sind nicht einheitlich.

Häufig wird eine initiale Therapie mit Penicillin G oder Ceftriaxon [15, 21, 25, 28] empfohlen und alternativ bei Betalaktamallergie Vancomycin [15, 21, 25] oder Clindamycin [15, 21, 25]. Für die orale Anschlusstherapie gibt es sowohl Daten, die keinen Vorteil einer Rifampicin Therapie zeigen [13], als auch Untersuchungen, die die Zugabe von Rifampicin unterstützen [11]. Wird nach Reimplantation einer neuen Prothese oder nach einem DAIR eine Therapie mit Rifampicin empfohlen, werden als Kombinationspartner in der Literatur z. B. Amoxicillin, Doxycyclin oder Levofloxacin gewählt [6, 25].

### Enterokokken

Enterokokken sind für 2,3–15 % der PPI verantwortlich und kommen regelmäßig als Teil polymikrobieller Infektionen vor [19, 26]. Sie gelten als weniger pathogen als andere grampositive Erreger wie beispielsweise *Staphylococcus aureus* oder *Streptococcus pyogenes* [17]. Gleichzeitig werden enterokokkenbedingte PPI teilweise als schwierig zu behandeln beschrieben [21]. Einige Autoren berichten abhängig von der chirurgischen Herangehensweise von relativ hohen Rezidivraten [8, 19, 29], wobei dieses Thema kontrovers diskutiert wird und zum Teil auch relativ hohe Heilungsraten publiziert wurden [9, 26]. Antibiotisch wird für die intravenöse Therapie beim Vorliegen von *Enterococcus faecalis* häufig eine Therapie mit Ampicillin in Kombination mit Ceftriaxon oder Gentamicin und zur Behandlung von *Enterococcus faecium* eine Therapie mittels Vancomycin empfohlen, wobei die Ergebnisse der Sensitivitätstestungen grundsätzlich Berücksichtigung finden müssen. Vor dem Einsatz von Gentamicin ist das Vorliegen einer „High-level“-Resistenz auszuschließen. Ceftriaxon kann bei Sensibilität gegenüber Ampicillin als Kombinationspartner ohne Wirksamkeitstestung angewendet werden [26]. Weitere intravenöse Therapieoptionen stellen Daptomycin in hoher Dosierung (10–12 mg/kg KG) oder Fosfomycin als Kombinationspartner dar. Zur oralen Antibiotikatherapie stehen Amoxicillin und Linezolid zur Verfügung. Teilweise wird auch Doxycyclin empfohlen [15, 26].

### Gramnegative Erreger

Gramnegative Erreger werden als ursächlich in 5–23 % der PPI beschrieben [27, 33]. Für die initiale intravenöse Therapie werden in der Regel ein Betalaktamantibiotikum (z. B. Piperacillin/Tazobactam i.v. oder Ceftriaxon i.v. oder Meropenem i.v. in Abhängigkeit von der Resistenzlage) oder Ciprofloxacin i.v./oral empfohlen [21, 33]. Auch eine initiale Kombination aus Betalaktamantibiotikum plus Ciprofloxacin ist in Empfehlungen beschrieben [25]. Häufig wird auch in der Praxis zunächst mit einem bakteriziden Betalaktamantibiotikum begonnen und dann auf Ciprofloxacin oralisiert [15]. Ciprofloxacin gilt bei fremdmaterialassoziierten Infektionen mit gramnegativen Erregern als biofilmaktiv. Die Wirksamkeit von Ciprofloxacin auf gramnegative Erreger in Zusammenhang mit biofilmassoziierten Infektionen wurde in vitro, im Tiermodell und auch in klinischen Studien untersucht [20, 27, 31]. Generell ist bei gramnegativen Erregern die zunehmende globale Resistenzentwicklung zu beachten. In Deutschland spielt aktuell bei der PPI vor allem die Resistenz gegenüber Ciprofloxacin eine Rolle. Aufgrund der dann fehlenden biofilmaktiven Substanz gelten diese Erreger als schwer zu therapieren und werden als „Problemerreger“ bezeichnet.

### Kulturnegativer PPI

Kulturnegative PPI stellen eine therapeutische Herausforderung dar. Sie machen je nach Studie 7–42 % aller PPI aus [22]. Bei eindeutiger Klinik und/oder Histologie muss auch ohne Nachweis eines Erregers von einer Infektion ausgegangen werden und eine entsprechende operative Versorgung sowie empirische antibiotische Behandlung eingeleitet werden [30].

Initial sollte eine breit wirksame empirische Therapie unter Berücksichtigung des bekannten Erregerspektrums und individueller Risikofaktoren erfolgen (s. oben). Bei gutem Ansprechen auf die kalkulierte intravenöse Therapie ist eine Umstellung auf eine kalkulierte orale Therapie möglich [16]. Vergleichende Studien zur Wirksamkeit einzelner oraler Substanzen liegen bisher nicht vor. In der Regel werden Substanzen mit Wirksamkeit gegen die häufigen Erregergruppen eingesetzt. Die Kombination eines Fluorchinolons und Rifampicin ist aufgrund der exzellenten Wirksamkeit gegen Staphylokokken sowie der breiten Aktivität gegenüber weiteren Erregern eine empfehlenswerte orale Option [25].

## Behandlungsdauer

**Abb. 1:**
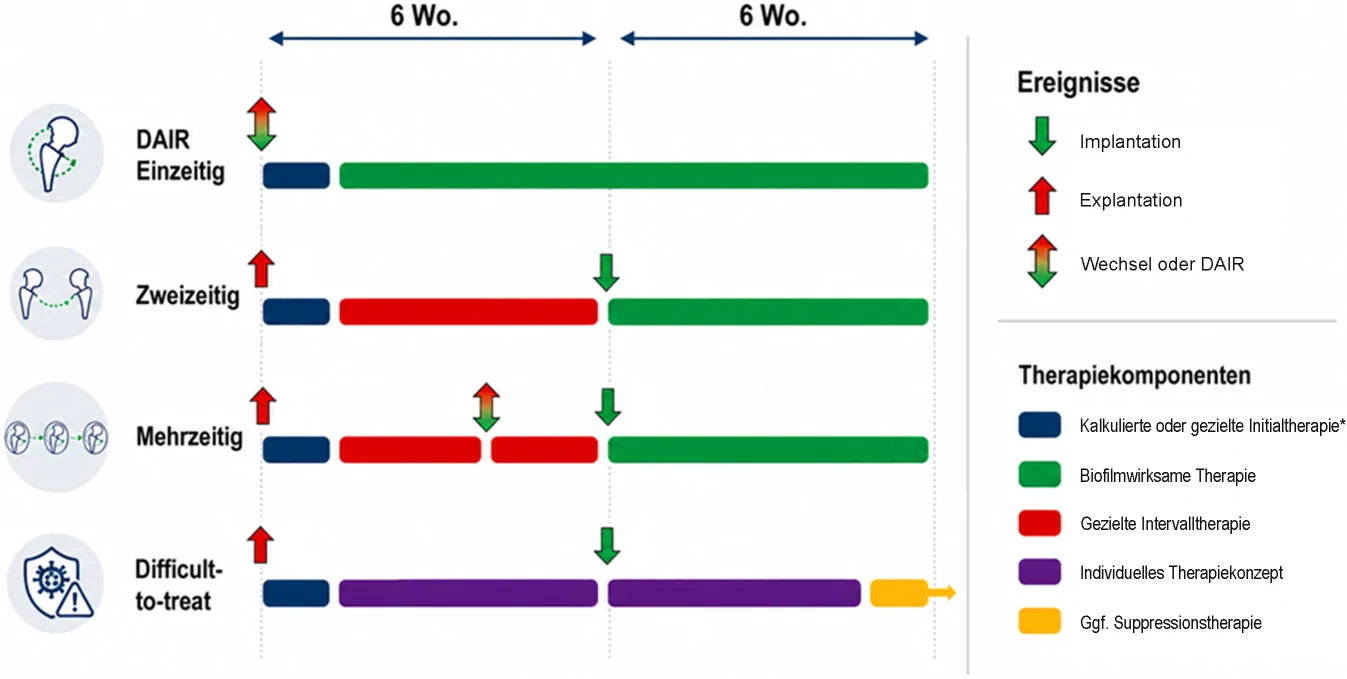
Therapiekonzepte bei periprothetischen Infektionen. *DAIR* Débridement, Antibiotikatherapie, Irrigation (Spülung) und „implantat retention“. Die chirurgische Intervention wird von einer initial intravenösen kalkulierten (Tab. 2) oder gezielten (Tab. 3) antiinfektiven Therapie begleitet, gefolgt von einer gezielten, teilweise oralen biofilmaktiven oder Intervalltherapie (Tab. 3). (Erstellt in PowerPoint, optisch optimiert mit Unterstützung von ChatGPT)

Die Behandlungsdauer bei PPI ist seit vielen Jahren Diskussionspunkt. In der Literatur sind Behandlungsdauern von 6 Wochen bis zu 6 Monaten beschrieben [15, 33]. Aktuell wird in den meisten Fällen in Deutschland insgesamt 12 Wochen therapiert, wobei in prothesenfreien Intervallen (ggf. unter liegendem Spacer) die biofilmaktive Substanz Rifampicin ausgespart bleibt. Typische Behandlungskonzepte werden in Abb. 1 zusammengefasst.

Eine Oralisierung erfolgt in der Praxis meist nach 7–14 Tagen. Dieses Vorgehen wurde kürzlich durch die OVIVA-Studie bestätigt [16]. Es konnte gezeigt werden, dass eine frühzeitige orale Antibiotikatherapie bei Knochen- und Gelenkinfektionen einer intravenösen Therapie hinsichtlich des Therapieversagens nach einem Jahr nicht unterlegen ist. Dabei wurden über 1000 Patienten randomisiert behandelt, einschließlich vieler mit implantatassoziierten Infektionen. Zudem war die orale Therapie mit weniger katheterassoziierten Komplikationen und geringeren Behandlungskosten verbunden. Bei der Oralisierung ist zu beachten, dass die Bioverfügbarkeit von intravenösen Antiinfektiva häufig nicht identisch mit der entsprechenden oralen Formulierung ist. Als Beispiel ist hier Cefuroxim zu nennen, welches i.v. gut wirksam ist, aber oral in diesem Kontext nicht verabreicht werden sollte.

Eine Suppressionstherapie nach vorheriger chirurgischer Keimlastreduktion sollte individuell im Einzelfall erwogen werden und kann über weitere 3 Monate bis hin zu einem Jahr oder in Einzelfällen lebenslang gegeben werden [12]. Hierbei sollten Substanzen gewählt werden, die besonders verträglich sind und Nebenwirkungen regelmäßig gemonitort werden. Abhängig von der zur Suppression eingesetzten Substanz und patientenindividuellen Faktoren wird eine Reduktion der Tagesdosis durchgeführt.

## Fazit für die Praxis

- Chirurgisches Vorgehen und antiinfektives Therapiekonzept stehen in direktem Zusammenhang und berücksichtigen patientenindividuelle und infektionsspezifische Gesichtspunkte.
- Als biofilmwirksame Substanz gilt Rifampicin als eines der wichtigsten Antibiotika bei der Behandlung von implantatassoziierten Infektionen mit Staphylokokken. Aufgrund der schnellen Resistenzentwicklung wird Rifampicin nie als Monotherapie, sondern immer mit einem geeigneten Kombinationspartner verabreicht. Zusätzlich müssen unter Rifampicin-Therapie unbedingt Nebenwirkungen/Interaktionen beachtet werden.
- Ciprofloxacin ist als biofilmaktiv bei implantatassoziierten Infektionen mit gramnegativen Erregern beschrieben. Beim Einsatz von Fluorchinolonen sind zahlreiche potenzielle Nebenwirkungen zu beachten (Rote-Hand-Brief).
- Die Therapiedauer beträgt in der Regel 12 Wochen und wird an die chirurgischen Konzepte angepasst.
- Ein enges Monitoring des Patienten zur Vermeidung von Nebenwirkungen, Wechselwirkungen und zum Erkennen von Rezidiven der Infektion ist essenziell. Eine enge Abstimmung von Klinik und ambulanten Strukturen, sowie Hausärzten bestimmt den Langzeiterfolg.
- Eine Langfassung der Handlungsempfehlung ist online verfügbar.

## Data Availability

Alle dieser Arbeit zugrunde liegenden Daten sind in diesem Artikel und im ausführlichen Literaturverzeichnis der Langfassung des Artikels enthalten.

## Abkürzungen

DAIR: Débridement, Antibiotikatherapie, Irrigation (Spülung) und „implant retention“
MRSA: Methicillin-resistenter *Staphylococcus aureus*
PPI: Periprothetische Infektion
